# Expression of DNA-damage response genes after exposure to high LET particles used in BNCT in glioblastoma cells with altered radiosensitivity

**DOI:** 10.1038/s41598-025-32635-1

**Published:** 2025-12-17

**Authors:** Martyna Araszkiewicz, Agnieszka Korgul, Katarzyna Tymińska, Urszula Kaźmierczak, Kinga Dyka, Patrycja Chuchała, Renata Grzela, Patrycja Kamińska, Roman Kuczma, Bohdan Paterczyk, Anna Stankiewicz-Drogoń, Beata Wielgus-Kutrowska, Agata Kustra, Michał Fryc, Piotr Bednarczyk, Kamila Maliszewska-Olejniczak

**Affiliations:** 1 Faculty of Physics, University of Warsaw, Warsaw, Poland; 2 National Centre for Nuclear Research, Otwock, Poland; 3 Department of Physics and Biophysics, Institute of Biology, Warsaw University of Life Sciences – SGGW, Warsaw, Poland; 4 Heavy Ion Laboratory, University of Warsaw, Warsaw, Poland; 5 Imaging Laboratory, Faculty of Biology, University of Warsaw, Warsaw, Poland

**Keywords:** Glioblastoma, DNA-PKcs, α particle irradiation, DNA damage response, Radiosensitivity, Cancer, Cell biology, Molecular biology, Oncology

## Abstract

DNA-dependent protein kinase catalytic subunit (DNA-PKcs) plays a central role in the repair of double-strand breaks (DSBs), but its deficiency alters the broader DNA damage response in glioblastoma cells exposed to α particle irradiation. Here, we investigated transcriptional changes in DNA repair pathways and cellular radiosensitivity in two isogenic glioblastoma cell lines differing in DNA-PKcs status: M059J (DNA-PKcs-deficient) and M059K (DNA-PKcs-proficient). Using pathway-focused qPCR, we profiled 30 genes involved in key DNA repair pathways and evaluated cell survival by clonogenic and MTT assays. M059J cells, despite DNA-PKcs deficiency, exhibited comparable survival fractions and higher metabolic activity than DNA-PKcs-proficient M059K cells following α particle irradiation. Irradiated M059J cells exhibited broad transcriptional upregulation of genes involved in double-strand break repair, single-strand break repair, mismatch repair, and nucleotide excision repair, reflecting compensatory activation of multiple repair mechanisms. In contrast, M059K cells displayed a restricted response, characterized primarily by strong *PRKDC* upregulation, the gene encoding DNA-PKcs. These findings highlight the pivotal role of DNA-PKcs status in shaping the DNA damage response and radiosensitivity of glioblastoma cells. Targeting compensatory repair pathways in DNA-PKcs-deficient tumors may offer novel strategies for radiosensitization in glioblastoma therapy.

**Supplementary Information:**

The online version contains supplementary material available at 10.1038/s41598-025-32635-1.

## Introduction

Glioblastoma multiforme (GBM) is the most common and aggressive primary brain tumor arising from astrocytes and is classified as a WHO grade 4 astrocytoma. Standard treatment includes surgical resection, chemotherapy with temozolomide, and radiation therapy, but the low survival rate of patients highlights the urgency for innovative and more effective therapeutic tools^1^. The response of this type of tumor to chemoradiotherapy is poor, possibly due to a higher repair activity of the genetic material, among other causes. DNA double-strand breaks (DSBs) are an essential type of lesion to the genetic material, which have the potential to trigger processes of cell death or cause gene aberrations that promote tumorigenesis. Radiotherapy eliminates tumor cells by causing DSBs. Therefore, targeting the cellular DNA damage response (DDR) is a promising strategy to enhance tumor radiosensitivity.

Both targeted alpha therapy and Boron Neutron Capture Therapy (BNCT) are currently under investigation as promising treatment options for brain tumors, including GBM. Radionuclides emitting high Linear Energy Transfer (LET) radiation, characterized by a short penetration range and potent cytotoxic effects, are increasingly applied in nuclear medicine to selectively eradicate malignant cells^2^. A prominent example of such an approach is targeted alpha therapy, wherein ^225^Ac-labeled radiopharmaceuticals are administered directly into the post-surgical tumor cavity to deliver localized, high-LET radiation to residual cancer cells, thereby minimizing damage to adjacent healthy tissues^3,4^. Beyond targeted alpha therapy, α particles also play a central role in BNCT, a treatment modality dedicated to tumors resistant to conventional therapies^5,6^. BNCT relies on the selective accumulation of boron-containing compounds, enriched with the stable^10^B isotope, within tumor cells. Following administration, the tumor is irradiated with a neutron beam of suitable energy, resulting in the capture of thermal neutrons by ^10^B nuclei. This reaction produces an unstable^11^B isotope that rapidly decays, releasing high-energy α particles and^7^Li nuclei. In Poland, preclinical studies on BNCT are underway at the National Centre for Nuclear Research in Świerk, where the MARIA research reactor serves as a source of thermal neutron beams for biological research^7^.

Alpha radiation, characterized by high LET, induces complex DNA damage, predominantly DSBs, which are more challenging to repair compared to damage caused by low LET radiation like X-rays or gamma rays^8–11^. The DNA damage response to alpha radiation involves several key mechanisms and pathways. Alpha particles have a high LET, meaning they deposit a significant amount of energy over a short distance, causing dense ionization tracks and complex DNA damage^6,8–10^. The damage includes DSBs, interstrand crosslinks, and clustered lesions, which are more difficult to repair than simple breaks^8–10^. The severe nature of the damage often leads to irreparable lesions, making alpha radiation highly effective in sterilizing cells^11^. Exposure to alpha radiation activates key DDR proteins such as ATM (Ataxia Telangiectasia Mutated), p53, and DNA-PKcs, which are crucial for initiating repair processes^9,12,13^. The primary repair mechanisms for DSBs include non-homologous end joining (NHEJ) and homologous recombination (HR). NHEJ is typically faster but more error-prone, while HR is more accurate but slower^12,13^. DDR activation leads to the engagement of cell cycle checkpoints, particularly at the G1/S and G2/M transitions, to allow time for repair before cell division^13^. When cells are exposed to both high LET (alpha particles) and low LET (X-rays or gamma rays), the combined effect can lead to more significant DNA damage and a more robust DDR than expected from the sum of individual exposures^8–10^. The sequence of exposure to different radiation types can influence DDR. For instance, alpha radiation followed by gamma radiation induces a stronger DDR compared to the reverse sequence^8^. Due to their high Relative Biological Effectiveness (RBE) and ability to cause irreparable damage, alpha particles are being explored for targeted radiotherapy, particularly in cancer treatment^11,14^. Alpha radiation can induce bystander effects, where non-irradiated cells exhibit damage responses due to signaling from irradiated cells, mediated by gap-junction communication^15^.

In glioblastoma cells, exposure to high LET α particles induces complex DNA damage, leading to a robust DNA damage response. This response involves the activation and upregulation of various DDR genes and proteins, which play crucial roles in DNA repair, cell cycle regulation, and apoptosis. Glioblastoma cells exhibit significant changes in the expression of DDR genes following high-LET radiation. For instance, genes involved in NHEJ - *XRCC5*, *XRCC6*, and HR pathways - *RAD51*, are upregulated^16–18^. Additionally, genes like *ATM*, *ATR*, and *CHK1*, which are critical for cell cycle checkpoint control and DNA repair, show increased expression^19,20^. High-LET radiation leads to extensive protein modifications and the activation of several transcription factors involved in DDR. This includes the phosphorylation of ATM, CHK2, and p53, which are essential for initiating DNA repair and apoptosis^19,21,22^. Glioblastoma cells with high levels of intrinsic chromosomal instability and impaired ATM signaling exhibit increased radioresistance to both low- and high-LET radiation. This suggests that the efficiency of DDR pathways significantly influences the survival and radiosensitivity of these cells^19,21,23^. The upregulation of DDR genes and proteins in response to high-LET radiation highlights potential targets for enhancing radiosensitivity. Inhibitors of DDR pathways, such as ATR and ATM inhibitors, have shown promise in increasing the radiosensitivity of glioblastoma cells to high-LET radiation^22^. Additionally, targeting specific DDR genes like *RAD18* and *DMC1* could further sensitize glioblastoma cells to radiation therapy^18,21^.

Understanding the expression and regulation of DDR genes in glioblastoma cells after high-LET α particle exposure is crucial for developing effective therapeutic strategies to overcome radioresistance and improve treatment outcomes.

## Materials and methods

### Cell culture

M059J (CRL-2366) and M059K (CRL-2365) cells were obtained from the American Type Culture Collection (ATCC), cultured according to the recommended conditions, and free of *Mycoplasma* contamination (Figure S1). The cells are glial in origin and were isolated from the brain tissue of a 33-year-old male patient diagnosed with malignant glioblastoma. These cell lines provide helpful model systems in which to study the role of DNA protein kinase in cellular and molecular processes involving DNA damage recognition and repair. The cells were cultured in a DMEM F12 medium with 10% FBS, penicillin, and streptomycin (10 mg/ml). Cells were cultured at 37 °C, 5% CO_2,_ and under 95% humidity. Growth media were changed according to the recommended conditions, and cells were passaged at 70–80% confluence using trypsin-EDTA in PBS. Cells were visualized under a Delta Optical IB-100 inverted microscope with Delta Optical DLT Cam Viewer software.

### *Mycoplasma* detection by PCR

Detection of *Mycoplasma* contamination in cell culture of M059J and M059K supernatants was performed using the EZ-PCR Mycoplasma Detection Kit (Biological Industries, Cat. No. 20-700-20) following the manufacturer’s instructions. This PCR-based assay targets conserved regions within the 16 S rRNA gene specific to *Mycoplasma* spp. For each sample, 1 ml of culture supernatant was transferred to a sterile 1.5 ml microcentrifuge tube and centrifuged at 250 × g for 1 min to remove cell debris. The clarified supernatant was then transferred to a new sterile tube and centrifuged at 15,000 × g for 10 min. The resulting pellet was resuspended in 50 µl of the supplied Buffer Solution and thoroughly mixed by pipetting. The suspension was then heated at 95 °C for 3 min to lyse the cells and release DNA. PCR reactions were set up in a 50 µl total volume using the components provided in the kit. Each reaction contained 10 µl of Reaction Mix, 5 µl of the prepared test sample, 1 µl of Internal Control DNA Template, 5 µl of Internal Control Primer Mix, and 29 µl of nuclease-free water (Ambion, Cat. No. AM9906). A positive control containing 1 µl of Positive Control DNA was included in a separate tube, as well as a negative control prepared with sterile distilled water. PCR amplification was carried out in a thermal cycler (T100 thermal cycler, Bio-Rad) under the following cycling conditions: initial denaturation at 94 °C for 30 s, followed by 35 cycles of denaturation at 94 °C for 30 s, annealing at 60 °C for 2 min, and extension at 72 °C for 1 min, then 94 °C 30 s, 60 °C 2 min, 72 °C 5 min, followed by a hold at 4 °C. PCR products were analyzed by agarose gel electrophoresis. A 2% agarose gel was prepared using Prona Standard Agarose (Bio-Standard) dissolved in 0.5× TBE buffer (Thermo Fisher Scientific, Cat. No. B52) and stained with SYBR Safe DNA Gel Stain (Invitrogen, Cat. No. S33102). Before loading, 15 µL of each PCR product was mixed with 6× loading dye (Thermo Fisher Scientific, Cat. No. R1161). A 1 kb DNA ladder, ready-to-use (Thermo Fisher Scientific, Cat. No. SM0313), was used as a molecular weight marker. DNA bands were visualized using the iBright Imaging System (Thermofisher Scientific). The presence of a 270 bp band indicated *Mycoplasma*-specific amplification, while a 357 bp band represented the internal control. Samples showing both bands were considered *Mycoplasma*-positive. Samples showing only the 357 bp band were considered negative.

### Characterization of ionizing radiation and exposure

Cell samples were irradiated using a specially designed irradiation setup consisting of a surface-mounted Am-241 source with an activity of 1.96 MBq, affixed to the inside of a Petri dish lid via a 6 μm-thick Mylar film^2^. The Am-241 source emits alpha particles with the following energy levels: 5388 keV (1.7%), 5443 keV (13.1%), 5485 keV (84.8%), and 5544 keV (0.4%) ^24^. The experimental setup maintained a precisely measured 6.8 mm air gap between the source and the biological sample. Before irradiation, cells were cultured on 30 mm diameter coverslips and then placed inside a sterile Petri dish for exposure. During irradiation, the Petri dish containing the cell samples was positioned beneath the α particle source to ensure uniform exposure to the radiation. To achieve different doses of α particle radiation, the exposure time was varied. The cell layer and medium height were determined based on measurements with a confocal microscope. The dose was calculated using the MCNP code^25^. Considering the precision of the measurements of individual geometry elements, we estimate that the uncertainty of 2 Gy is 1 Gy, which corresponds to an overall uncertainty of 50%.

### MTT assay

The viability of M059J and M059K cells was assessed using the MTT (3-(4,5-dimethylthiazol-2-yl)-2,5-diphenyl-2 H-tetrazolium bromide) assay, as previously described with some modifications^26,27^. Briefly, cells were seeded on 30 mm diameter coverslips placed in a 6-well plate at a density of 5 × 10⁴ cells per well. After 48 h, the cells were irradiated with doses of 0.08 Gy, 0.17 Gy, 0.33 Gy, 0.5 Gy, 0.67 Gy, 1.33 Gy, 2 Gy, and 2.67 Gy of alpha radiation. At 48 h post-irradiation, the medium was removed, and the cells were incubated with 1760 µl of fresh medium containing 0.5 mg/ml MTT for 4 h. Then the medium was removed, and formazan crystals were washed with 100 µl of PBS. Subsequently, 1000 µl of isopropanol was added to solubilize the formazan crystals for 15 min. The solubilized solution was then transferred to a 96-well plate (100 µl per well). Absorbance was measured at 590 nm using a microplate reader (Synergy H1MFDG Microplate Reader (BioTek) with Gen5 software). Results were expressed as a percentage relative to unirradiated control cells. Each group consisted of eight wells, and average values were calculated accordingly. All experiments were performed using different cell batches and were repeated at least three times.

### The clonogenic assay

The clonogenic assay (colony formation assay) was determined as described previously with some modifications^28,29^. Cells were seeded out in numbers increasing with the radiation dose. For the M059J cell line, 600 unirradiated cells were seeded, while growing numbers were used for higher doses: 900 cells for 0.33 Gy, 1200 cells for 0.67 Gy, 1700 cells for 1 Gy, 2000 cells for 1.33 Gy, 3000 cells for 1.67 Gy, and 4000 cells for 2 Gy. Similarly, for the M059K cell line, 1500 unirradiated cells were seeded, with 2000 cells for 0.67 Gy, 3500 cells for 1.33 Gy, 4500 cells for 2 Gy, and 5500 cells for 2.67 Gy. Cells were cultured on a 9 cm Petri dish with 2 ml DMEM F12 medium and incubated for up to 12 days at 5% CO_2_ and 37 °C. After incubation, the medium was removed, the cells were washed with PBS, and fixed with methanol for 10 min. Cells were stained with 20% Giemsa solution for 15 min., immersed in tap water, and dried at room temperature. The number of colonies composed of over 50 cells was observed using a 10x objective under a Delta Optical IB-100 inverted microscope and counted using a YOLOv5-based pipeline with sliding window inference^30,31^. Clone formation efficiency (plating efficiency, PE) was determined using the equation: (clone number/plated cell number) × 100%. The survival fraction (SF) was determined according to the equation: (PE of treated cells/PE of untreated cells) × 100% ^28^.

### Immunofluorescence detection of γ-H2AX foci in M059J and MO59K cell lines

15 min after irradiation of M059J and M059K cells were fixed in cold methanol (− 20 °C) for 5 min, followed by rehydration in PBS (4 °C) for 3 × 10 min. To block non-specific binding, cells were incubated overnight in blocking buffer consisting of 2% BSA, 10% dried milk powder, and 1.0% Triton X-100, prepared in KCM buffer (120 mM KCl, 20 mM NaCl, 10 mM Tris·HCl, 1 mM EDTA, 1.0% Triton X-100, pH 8.0). Samples were then incubated for 2 h with anti-p-H2A.X (Ser139), clone JBW301, Alexa Fluor 488-conjugated (1:400; Sigma-Aldrich, St. Louis, MO, USA) diluted in blocking buffer, and subsequently washed in KCM buffer (three times, 15 min each). Afterward, cells were incubated with Alexa Fluor 488 goat anti-mouse IgG (H + L) secondary antibody (Invitrogen, Eugene, OR, USA) in blocking buffer for 1 h at room temperature. Final washes were performed in PBS (4 × 10 min), and cells were mounted using Vectashield Antifade Mounting Medium with DAPI (Vector Laboratories, Burlingame, CA, USA). Confocal Z-stack images were acquired in .nd2 format using a Nikon A1R MP multiphoton confocal microscope. Z-stacks were collected at 1 μm intervals, and image analysis was performed using NIS-Elements Viewer Software and ImageJ. The set of lasers and filters used in the specific channels was as follows: blue channel (nuclei): Excitation wavelength (Ex) 404 nm; Emission wavelength (Em) 425–475 nm; green channel (γH2AX foci): Ex 488 nm; Em 500–550 nm. To achieve higher-resolution 3D imaging, the Z-step was reduced from 1 μm to 0.105 μm. For subsequent quantitative analysis, 3D Z-stack images were converted into 2D projections using the ‘Sum Slices’ function in ImageJ, which generates a single 2D image by summing the corresponding pixel intensities across all selected slices. Foci were then quantified using FociCounter, with identical counting parameters applied across all samples^32^.

### RNA isolation and cDNA synthesis

Total RNA was isolated from cells cultured on 30 mm diameter coverslips using an RNeasy Mini Kit (Qiagen, TX, USA) according to the manufacturer’s instructions with DNase I (Qiagen) treatment as previously published^28,29^. RNA concentrations and purity were measured using µDrop™ Duo Plates in a Multiskan SkyHigh microplate reader (Thermo Scientific, DE, USA). The RNA (1 µg) was subjected to reverse transcription using an iScript cDNA Synthesis Kit (Bio-Rad) with RNase H^+^ MMLV reverse transcriptase according to the producer’s protocol.

### qPCR assay

The expression of selected 30 genes from DNA damage and DNA repair signaling pathways (listed in alphabetical order: *ATM*, *ATR*, *BRCA1*, *BRCA2*, *CHEK1*, *CHEK2*, *ERCC1*, *H2AFX*, *LIG1*, *LIG3*, *LIG4*, *MRE11A*, *MSH2*, *MSH3*, *NBN*, *OGG1*, *PARP1*, *PARP2*, *PARP3*, *PRKDC*, *RAD50*, *RAD51*, *RAD54L*, *TP53BP1*, *XRCC1*, *XRCC2*, *XRCC3*, *XRCC4*, *XRCC5*, *XRCC6*) was screened with Prime PCR assay designed custom SYBR plate (Bio-Rad Laboratories, Inc.) according to the manufacturer’s protocol (Bio-Rad Laboratories, Inc.) and as published^28^. As the reference housekeeping gene, *ACTB* (encoding beta-actin) was selected. The reaction was performed using 1 µl of cDNA sample, iTaq™ Universal SYBR^®^ Green Supermix (Bio-Rad), and 20xPrime PCR assay primers dried in wells of a 96-well plate according to the manufacturer’s protocol for a custom plate (SYBR Green^®^ reaction setup). All qPCR experiments were performed using a CFX Opus 96 Real-Time PCR System (Bio-Rad). Gene expression data were analyzed using CFX Manager™ Software (Bio-Rad). Fold change was calculated using the standard Eq. 2^−(ΔΔCt)^ as described previously^28^. The expression of each gene was calculated based on three biological replicates.

### Statistical analysis

All experiments were performed in three independent biological replicates to confirm reproducibility. Results were displayed as mean ± SD (clonogenic assay and MTT) or mean ± SEM (qPCR). One-way ANOVA was used to analyze experimental data. P-values were considered significant: *p ≤ 0.05, **p ≤ 0.01, ***p ≤ 0.001, ****p ≤ 0.0001, ns – not significant.

## Results

### Alpha particle irradiation reduced clonogenic survival and metabolic activity in M059J and M059K glioblastoma cells

**Fig. 1:**
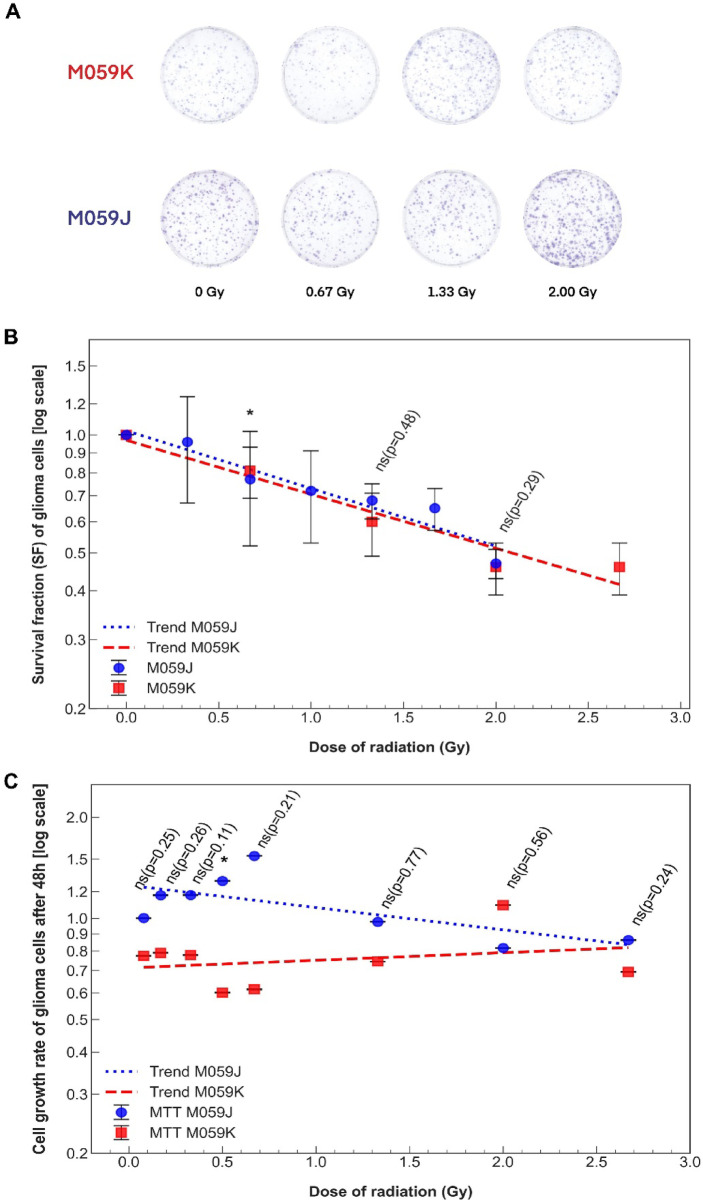
Clonogenic survival and growth rate of M059J and M059K glioblastoma cells after α particle irradiation. (**A**): Representative images of colony formation in M059J and M059K cells irradiated with α particles at doses of 0, 0.67, 1.33, and 2 Gy. Colonies were stained with 20% Giemsa solution after 12 days of incubation. (**B**): Clonogenic survival fraction (SF) plotted on a logarithmic scale for irradiation doses ranging from 0 to 2.67 Gy (0, 0.33, 0.67, 1.00, 1.33, 1.67, 2.00 for M059J; 0, 0.67, 1.33, 2.00, and 2.67 Gy for M059K). **(C)**: MTT assay showing metabolic activity measured 48 h post-irradiation at doses of 0.08, 0.17, 0.33, 0.5, 0.67, 1.33, 2.00, and 2.67 Gy. Error bars represent standard deviations (SD) of three independent experiments. Uncertainties in cell growth rate are smaller than the symbol size and are therefore not visible. Statistical significance was determined by one-way ANOVA (*p ≤ 0.05, **p ≤ 0.01, ***p ≤ 0.001, ****p ≤ 0.0001, ns – not significant).

To investigate the impact of DNA-PKcs status on glioblastoma cell survival after α particle irradiation, we assessed both long-term clonogenic survival and short-term MTT metabolic activity in M059J (DNA-PKcs-deficient) and M059K (DNA-PKcs-proficient) cells across a range of doses (0–2.67 Gy). Based on the clonogenic assay, both cell lines displayed a dose-dependent reduction in survival fraction (SF) (Fig. 1A and B). At lower doses (0.33–1.33 Gy), M059K cells showed a tendency toward reduced survival compared to M059J cells, suggesting greater initial radiosensitivity. For example, at 1.33 Gy, the SF values were approximately 0.68 ± 0.07 for M059J and 0.60 ± 0.11 for M059K. At 0.67 Gy, survival was 0.77 ± 0.25 for M059J and 0.81 ± 0.12 for M059K, indicating similar clonogenic potential at lower doses. At the dose of 2 Gy, survival values converged, with M059J at 0.47 ± 0.04 and M059K at 0.46 ± 0.07, indicating that high-LET α particle irradiation led to substantial cytotoxicity in both lines. To validate these observations, we performed an MTT assay, which confirmed the differential sensitivity between the cell lines at lower doses (Fig. 1C). At 0.67 Gy, M059J cells displayed increased metabolic activity (1.533 ± 0.002), while M059K cells exhibited markedly lower viability (0.615 ± 0.002), suggesting higher short-term sensitivity of DNA-PKcs-proficient cells at low-dose irradiation. This trend persisted up to ~ 1.33 Gy, where M059K cells consistently exhibited lower metabolic activity compared to M059J. At 2 Gy, both cell lines showed reduced metabolic activity, although to different extents: M059J cells reached 0.816 ± 0.001, whereas M059K cells maintained a higher metabolic level of 1.095 ± 0.001, potentially reflecting compensatory responses despite similar survival outcomes. The clonogenic and MTT assays demonstrate that DNA-PKcs-proficient M059K cells are initially more sensitive to α particle-induced cytotoxicity, while M059J cells retain higher survival and metabolic activity at early dose points. The convergence of survival at higher doses suggests that extensive DNA damage may ultimately overwhelm the DNA repair capacity of both cell lines, irrespective of DNA-PKcs status. Based on the clonogenic and MTT assays, a dose of 2 Gy was selected as it induced significant but not complete cytotoxicity in both cell lines, allowing for the assessment of DNA damage responses. This dose enabled the analysis of active transcriptional changes without causing excessive cell death that could compromise gene expression profiling.

### γH2AX foci formation in M059J and M059K cells following irradiation

**Fig. 2:**
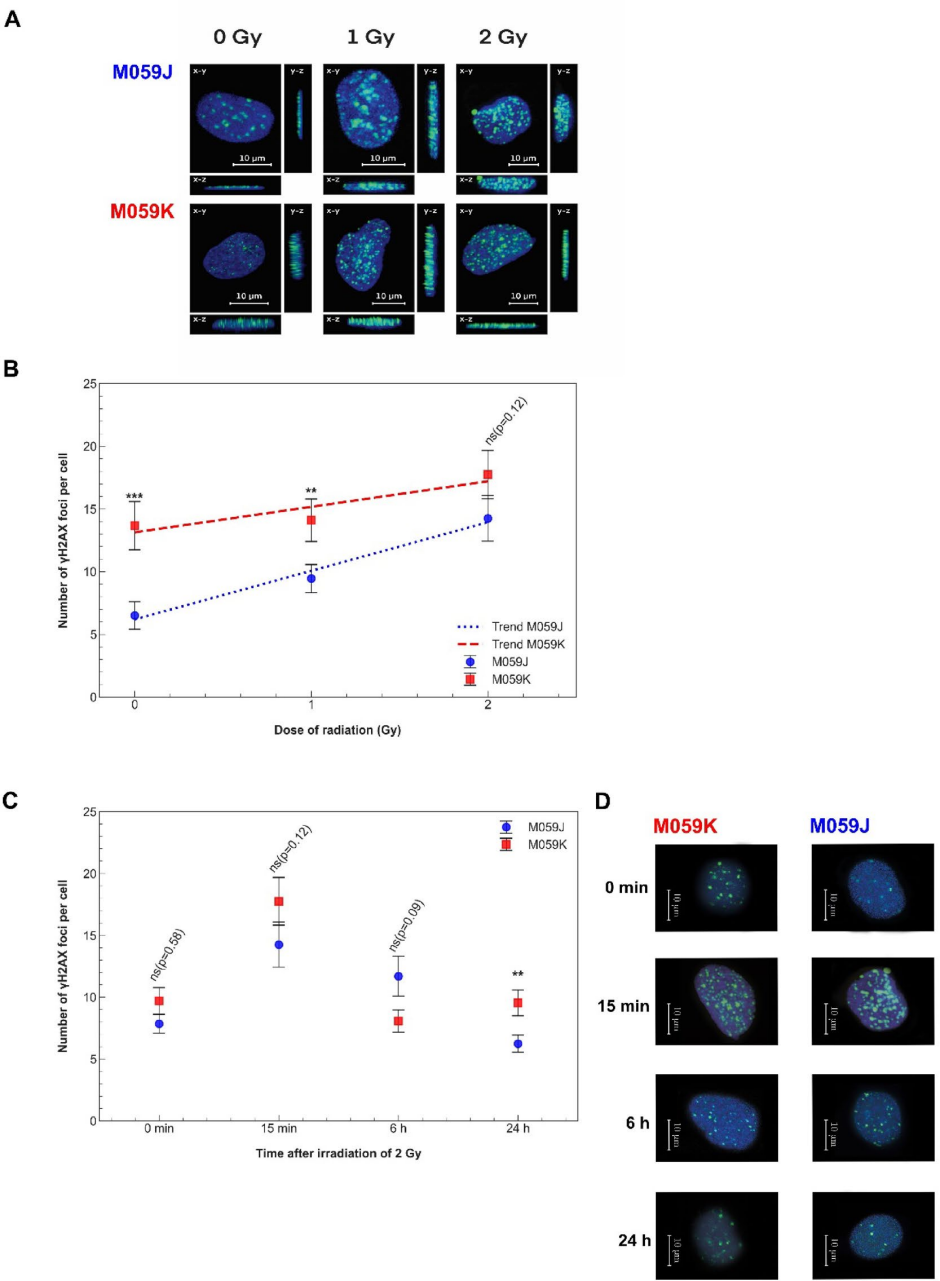
γH2AX foci formation in M059J and M059K cells following α-irradiation. (**A**): Representative confocal micrographs showing γH2AX foci (green, Alexa Fluor 488) and nuclei (blue, DAPI) in M059J and M059K glioblastoma cells 15 min after exposure to 1 Gy, and 2 Gy α-particle irradiation (0 Gy – control samples). Each nucleus was visualized in 3D and projected onto the **x**–y, x–z, and y–z planes. Representative nuclei were randomly selected and imaged at higher resolution, which was achieved by reducing the Z-step from 1 μm to 0.105 μm using a Plan Apo VC 60×/1.4 oil immersion objective. Scale bar = 10 μm. Images were acquired under identical confocal settings (laser power, detector gain, and exposure) across all groups. (**B**): Dose–response curves of γH2AX foci per cell in M059J and M059K glioblastoma cells exposed to increasing doses of α-particle irradiation. Data are expressed as mean ± SEM from three independent experiments (≥ 100 nuclei analyzed per condition) and were generated using the FociCounter programme. Statistical significance was determined by one-way ANOVA (*p ≤ 0.05, **p ≤ 0.01, ***p ≤ 0.001, ****p ≤ 0.0001, ns – not significant). (**C**): yH2AX foci formation and loss (DSB repair dynamics) upon exposure to 2 Gy of α-particle irradiation for time points: 0 min, 15 min, 6 h, and 24 h – a graph representing yH2AX foci per cell in M059J and M059K cell lines. Data are expressed as mean ± SEM from three independent experiments (≥ 100 nuclei analyzed per condition) and were generated using the FociCounter programme. Statistical significance was determined by one-way ANOVA (*p ≤ 0.05, **p ≤ 0.01, ***p ≤ 0.001, ****p ≤ 0.0001, ns – not significant). (**D**): Representative images of the analyzed cells for each time point: yH2AX (green, Alexa Fluor 488), nuclei (blue, DAPI).

To assess the DNA damage response induced by ionizing radiation, we performed immunofluorescence staining for γH2AX, a well-established marker of DNA double-strand breaks, in two glioblastoma cell lines: M059J and M059K. Cells were exposed to 0 Gy (control) and 2 Gy, and stained for γH2AX (green) and DAPI (blue) to visualize nuclear morphology. In M059J cells (Fig. 2A), the γH2AX signal was minimal under basal conditions (0 Gy). Upon irradiation with 2 Gy, a robust increase in γH2AX foci was observed, indicating the formation of DSBs. The signal was localized exclusively to the nuclei, confirming radiation-induced DNA damage. A similar pattern was observed in M059K cells (Fig. 2A). Under control conditions, low-level γH2AX staining was present, suggesting minimal spontaneous DNA damage. Following 2 Gy exposure, an increase in nuclear γH2AX foci was detected compared to the non-irradiated control. In M059J cells, exposure to 1 Gy and 2 Gy α-particle irradiation resulted in the formation of large and intense γH2AX foci, markedly increased compared to non-irradiated controls (Fig. 2A). These radiation-induced foci (RIF) were not only more numerous but also appeared enlarged, consistent with the accumulation of complex DNA double-strand breaks and slower repair kinetics. By contrast, M059K cells also exhibited an increase in γH2AX foci after irradiation, but the foci were generally smaller, more evenly distributed, and less pronounced than in M059J cells. Quantitative analysis confirmed a significant dose-dependent increase in the number of γH2AX foci per nucleus in both cell lines (Fig. 2B), with consistently higher values observed in M059K, whereas the foci in M059J tended to be larger and more clustered.

In the M059J cell line, the mean number of γH2AX foci in the control was 6.50 ± 1.10 (Fig. 2B). In response to 1 Gy, it increased to 9.45 ± 1.12, and after 2 Gy, it was 14.25 ± 1.82. The relative increase in the mean number of foci, normalized to the control, was 45% for 1 Gy and 119% for 2 Gy, indicating a significant rise in foci formation at 2 Gy compared to the control. A similar, though slightly lower, increase was seen in the M059K cell line, with 3% after 1 Gy and 27% after 2 Gy (Fig. 2B). The mean numbers of γH2AX foci in M059K were 13.67 ± 1.92 for the control, 14.10 ± 1.69 after 1 Gy, and 17.74 ± 1.93 after 2 Gy. Confocal microscopy and quantitative analysis demonstrated a dose-dependent induction of γH2AX foci in both glioblastoma cell lines, indicating that while M059K cells accumulate higher numbers of foci, M059J cells develop larger radiation-induced foci, consistent with impaired DNA repair capacity due to DNA-PKcs deficiency.

To characterize the dynamics of radiation-induced DNA double-strand break signalling, we quantified γH2AX foci in M059J and M059K cells at four time points following exposure to 2 Gy (Fig. 2C and D). At 0 min, the basal levels of γH2AX foci were comparable between M059J and M059K, with values of 7.84 ± 0.76 and 9.70 ± 1.07, (*p* = 0.58). At 15 min post-irradiation, both lines exhibited a pronounced rise in γH2AX foci (14.25 ± 1.82 in M059J and 17.74 ± 1.93 in M059K), consistent with the rapid formation of DSB-associated chromatin marks, and the difference between M059J and M059K (*p* = 0.12). By 6 h, γH2AX foci declined in both lines (11.69 ± 1.62 in M059J and 8.06 ± 0.90 in M059K), (*p* = 0.09). A clear divergence between the two cell lines emerged at 24 h post-irradiation. While M059J cells showed a marked reduction in residual γH2AX foci (6.24 ± 0.70), M059K cells retained substantially higher levels (9.53 ± 1.04), resulting in a statistically significant difference (*p* < 0.01). Between 15 min and 6 h post-irradiation, M059K cells exhibited a more rapid decline in γH2AX foci than M059J, consistent with more efficient DSB repair.

### Expression of DNA-damage response and repair genes after exposure to α-radiation in glioblastoma cells with altered radiosensitivity

To investigate the transcriptional regulation of DNA repair pathways in glioblastoma cells with different DNA-PKcs status, we analyzed the expression of key DNA repair genes in M059J and M059K cell lines under basal conditions and following α particle irradiation. Consistent with their known genetic background, qPCR confirmed the absence of *PRKDC* expression in M059J and strong expression in M059K under basal conditions (Figure S2), validating the DNA-PKcs–deficient versus proficient status of these lines. Next, the experimental design included four comparative analyses to assess both constitutive gene expression differences and irradiation-induced transcriptional responses. First, we compared the basal gene expression between the two cell lines (M059J vs. M059K, non-irradiated) to identify constitutive differences associated with DNA-PKcs deficiency. Second, we evaluated the effect of α particle exposure within each cell line separately: M059J irradiated with 2 Gy versus M059J non-irradiated (M059J 2 Gy vs. J) and M059K irradiated with 2 Gy versus M059K non-irradiated (M059K 2 Gy vs. K). Finally, we compared the transcriptional responses of both irradiated cell lines (M059J vs. M059K, both irradiated with 2 Gy) to identify differences in their responses to DNA damage. The genes selected for this analysis represent key components of major DNA repair pathways. The nucleotide excision repair (NER) pathway was represented by *ERCC1*. The mismatch repair (MMR) pathway includes *MSH2* and *MSH3*. The single-strand break repair (SSBR) and base excision repair (BER) pathways were represented by *LIG1*, *LIG3*,* OGG1*, *PARP1*, *PARP2*, and *XRCC1*. The most extensive group comprised genes involved in double-strand break repair (DSBR), encompassing both damage sensors, transducers, and effector proteins: *ATM*, *ATR*, *BRCA1*, *BRCA2*, *CHEK1*, *CHEK2*, *H2AFX*, *LIG4*, *MRE11A*, *NBN*, *PARP3*, *PRKDC*, *RAD50*, *RAD51*, *RAD51L*, *TP53BP1*, *XRCC2*, *XRCC3*, *XRCC4*, *XRCC5*, and *XRCC6*.

### Constitutive differences in DNA repair gene expression between M059J and M059K glioblastoma cell lines under basal conditions

**Fig. 3:**
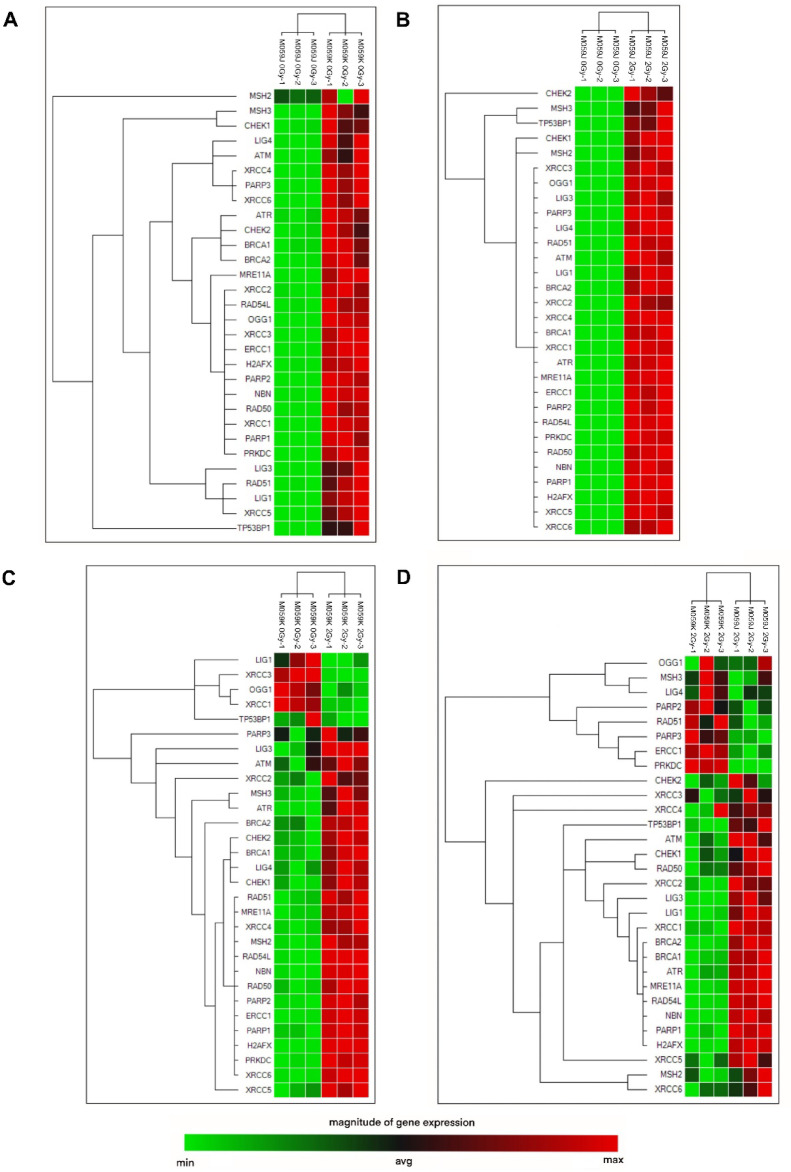
2-D clustergram heatmap analysis of the expression of DNA-damage signaling genes in glioma cells after α-irradiation (2 Gy). (**A**): M059J 0 Gy vs. M059K 0 Gy; (**B**): M059J 2 Gy vs. M059J 0 Gy; (**C**): M059K 2 Gy vs. M059K 0 Gy; (**D**): M059J 2 Gy vs. M059K 2 Gy. The red color characterizes a relatively high level of gene expression, whereas the green color indicates a low level. The data were analyzed (*n* = 3) and clustered by targets using the Reference Gene Selection Tool from CFX Maestro Software v2.3 (Bio-Rad Laboratories, Inc.).

**Fig. 4:**
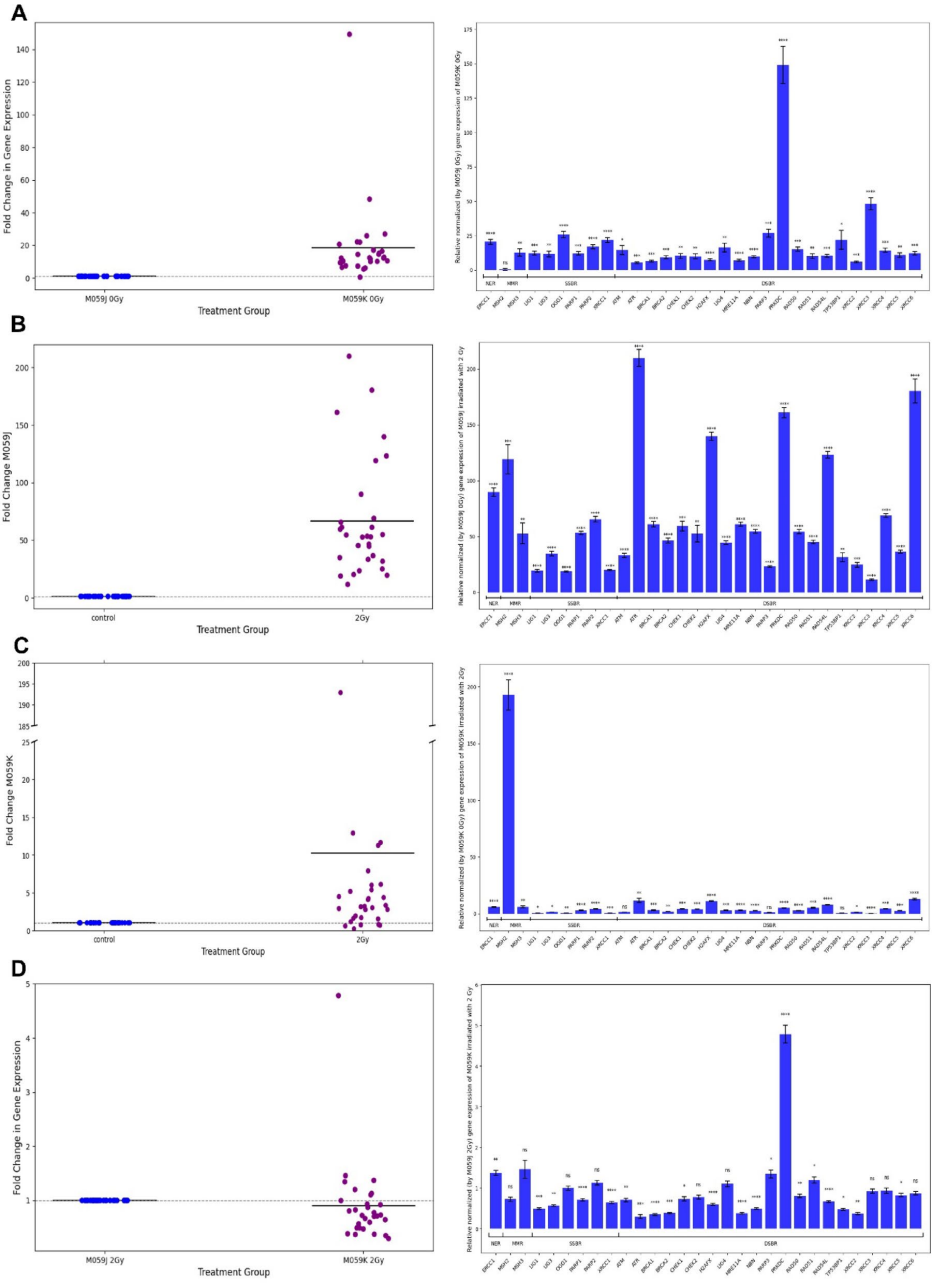
Relative to control, the normalized expression of DNA-damage response and repair genes after α-irradiation (2 Gy). (**A**): M059J 0 Gy vs. M059K 0 Gy; (**B**): M059J 2 Gy vs. M059J 0 Gy; (**C**): M059K 2 Gy vs. M059K 0 Gy; (**D**): M059J 2 Gy vs. M059K 2 Gy. Left panels: fold change in overall gene expression, symbols are mean gene expression for each of the 30 DNA-damage signaling genes included on the designed 96-well PCR arrays. Right panels: bar plot of the relative expression of all genes calculated *via* the ΔΔCt method. The bars correspond to the mean ± SEM (*n* = 3). One-way ANOVA was used to analyze experimental data. *P*-values were considered significant: *p ≤ 0.05, **p ≤ 0.01, ***p ≤ 0.001, ****p ≤ 0.0001, ns – not significant.

To assess the basal activity of DNA repair pathways in glioblastoma cell lines with differing DNA-PKcs status, we compared the expression levels of key DNA repair genes in untreated (0 Gy) M059J and M059K cells. Relative gene expression was normalized to the M059J cell line. The most pronounced difference in basal gene expression between M059K and M059J cells was observed for *PRKDC*, which was expressed approximately 150-fold higher in M059K cells compared to M059J, confirming the DNA-PKcs-proficient status of this line (Figs. 3A and 4A). In addition, *XRCC3*, a key homologous recombination gene belonging to the DSBR pathway, showed a ~ 50-fold higher expression in M059K cells, while *PARP3* (SSBR/DSBR) and *OGG1* (SSBR) were expressed ~ 25-fold higher in M059K cells. These results indicate that, under basal conditions, M059K cells exhibit overall higher expression of all DNA repair genes tested, compared to DNA-PKcs-deficient M059J cells. This suggests that M059K cells maintain a constitutive activation of multiple DNA repair pathways, potentially reflecting their greater repair capacity.

### Transcriptional activation of multiple DNA repair pathways in M059J cells after α particle irradiation

In the second comparison group, we analyzed the changes in gene expression in DNA-PKcs-deficient M059J cells exposed to 2 Gy of α particle irradiation compared to non-irradiated cells. The expression of all analyzed DNA repair genes in M059J cells was markedly upregulated compared to non-irradiated control (Figs. 3B and 4B). This analysis revealed a broad and strong transcriptional activation of multiple DNA repair pathways. The highest induction was observed for *ATR*, which increased by approximately 220-fold, reflecting robust activation of DNA damage checkpoint signaling (Fig. 4B, right panel). *XRCC6* showed an ~ 180-fold upregulation, suggesting compensatory activation of DNA end-joining components in the absence of DNA-PKcs. *PRKDC*, although non-functional in M059J, was also transcriptionally induced by ~ 150-fold, indicating an attempt to activate the classical NHEJ pathway at the mRNA level. Additionally, *H2AFX* was upregulated by ~ 140-fold, consistent with enhanced DNA damage signaling, while homologous recombination repair factors such as *RAD54L* (~ 130-fold) and mismatch repair component *MSH2* (~ 120-fold) were also strongly induced. Other genes, such as *ERCC1*, involved in nucleotide excision repair, showed a more moderate increase of ~ 90-fold, whereas *MSH3*, another mismatch repair gene, was upregulated by ~ 50-fold. These findings demonstrate that α particle irradiation induces a broad transcriptional response in M059J cells, engaging checkpoint kinases, homologous recombination, DNA end-joining components, and mismatch and nucleotide excision repair pathways, reflecting a compensatory adaptation to DNA-PKcs deficiency.

### Transcriptional response of DNA repair genes in M059K cells following α particle irradiation

In the third comparison group, we analyzed transcriptional changes in DNA-PKcs-proficient M059K cells irradiated with 2 Gy relative to non-irradiated controls. The analysis revealed a much narrower transcriptional response compared to DNA-PKcs-deficient M059J cells, with only a few genes showing induction (Figs. 3C and 4C). The most prominent increase was observed for *MSH2*, a mismatch repair gene, which showed an approximately 200-fold upregulation, indicating that mismatch repair was the most strongly activated pathway in M059K cells following irradiation (Fig. 4C, right panel). *XRCC6*, encoding Ku70, was the second most strongly induced gene (~ 30-40-fold increase), suggesting reinforcement of the classical non-homologous end joining pathway. Checkpoint kinase *ATR* was moderately upregulated (~ 20-30-fold), indicating activation of DNA damage checkpoint signaling. In addition, *H2AFX* (~ 15-20-fold) and *RAD54L* (~ 10-15-fold), both involved in DNA damage signaling and homologous recombination, were significantly induced. Other DNA repair genes, including *ERCC1*, *XRCC4*, *RAD50*, *PRKDC*, and *CHEK2*, showed only modest increases (~ 5- to 10-fold). Most of the remaining genes displayed minimal or moderate transcriptional changes, suggesting that M059K cells relied predominantly on their pre-existing DNA repair capacity rather than a broad transcriptional activation of DNA repair pathways. These results demonstrate that in response to α particle irradiation, DNA-PKcs-proficient M059K cells primarily activate mismatch repair and non-homologous end joining components, with limited induction of homologous recombination and single-strand break repair genes. This restricted transcriptional response likely reflects the sufficient basal activity of DNA-PKcs-dependent repair mechanisms.

### Differential expression of selected DNA repair genes between irradiated M059J and M059K cells

In the fourth comparison group, where both M059J and M059K cells were irradiated with 2 Gy, only a few DNA repair genes showed notable differences in expression between the cell lines (Figs. 3D and 4D). As expected, the most pronounced difference was observed for *PRKDC*, whose expression remained significantly higher in M059K cells (~ 5-fold increase compared to M059J), confirming the DNA-PKcs-proficient phenotype of M059K even after irradiation (Fig. 4D, right panel). Additionally, *MSH3*, a component of the mismatch repair pathway, exhibited moderately higher expression in M059K cells (~ 2-fold increase), suggesting a greater activation of mismatch repair in DNA-PKcs-proficient cells under DNA damage stress. *ERCC1*, involved in nucleotide excision repair, was also upregulated in M059K compared to M059J, though to a lesser extent (~ 1.5-fold higher expression). These findings indicate that, after α particle irradiation, most DNA repair genes are similarly induced in both cell lines, while key differences in *PRKDC*, *MSH3*, and *ERCC1* reflect the underlying repair pathway capacities shaped by DNA-PKcs proficiency.

## Discussion

M059J and M059K are human glioblastoma cell lines that differ in their DNA-PKcs activity, which plays a fundamental role in the repair of DSBs. M059J cells are DNA-PKcs-deficient, whereas M059K cells possess functional DNA-PKcs, allowing them to repair DSBs efficiently. This difference profoundly influences their cellular response to α particle irradiation, which induces complex DNA lesions^33^. Interestingly, inhibition of DNA-PKcs in glioblastoma cells reduces radiation-induced angiogenesis, migration, and invasion, suggesting that DNA-PKcs suppression could enhance radiotherapeutic efficacy by hindering tumor progression^34^. Our study highlights fundamentally different transcriptional responses to DNA damage in DNA-PKcs-deficient M059J versus DNA-PKcs-proficient M059K glioblastoma cells. The lack of *PRKDC* expression in M059J confirms DNA-PKcs deficiency stemming from a frameshift mutation in *PRKDC*^35^. Complementation studies of M059J with *PRKDC* restored DNA-PKcs activity and radioresistance, affirming DNA-PKcs as the primary defect in these cells^36^. Under basal conditions, DNA-PKcs-proficient M059K cells exhibited significantly higher expression of multiple DNA repair genes, particularly *PRKDC*, *XRCC3*, *PARP3*, and *OGG1*, suggesting constitutive activation of double-strand and single-strand break repair pathways. This likely reflects an overall greater intrinsic DNA repair capacity, consistent with previous studies showing constitutive activation of DNA repair in tumors with intact DNA-PKcs. Previous functional studies showed that M059J cells are approximately 30-fold more radiosensitive than M059K, reflecting their impaired DSB repair due to DNA-PKcs deficiency^37^. Complementing these genomic data, earlier biochemical evidence has demonstrated slower repair kinetics and accumulation of DNA damage in M059J compared to M059K^37,38^.

In contrast, DNA-PKcs-deficient M059J cells, which lack functional NHEJ, exhibited low basal expression of most DNA repair genes but responded to α particle irradiation with robust transcriptional activation across multiple pathways. The strongest induction was observed for *ATR*, *XRCC6*, *H2AFX*, and *RAD54L*, reflecting strong activation of checkpoint signaling, alternative end-joining, and DNA damage sensors, consistent with compensatory activation of homologous recombination and checkpoint pathways. In addition, previous studies have demonstrated that M059J cells, lacking DNA-PKcs activity, undergo pronounced cell cycle G2/M-phase arrest after irradiation^37,39^. Despite the absence of DNA-PKcs, M059J cells can still activate the G2/M checkpoint. This is likely due to the involvement of other checkpoint proteins such as Chk1 and Chk2, which can compensate for the lack of DNA-PKcs^40,41^. Thus, the strong transcriptional activation we observed in M059J may also be partly explained by checkpoint-driven cell cycle redistribution, in agreement with these published reports. ATR (Ataxia Telangiectasia and Rad3-related protein) is crucial for the DNA damage response, particularly in recognizing DNA damage induced by ionizing radiation and activating downstream pathways for cell cycle arrest and DNA repair^42^. This activation leads to phosphorylation of key proteins involved in DNA repair and cell cycle regulation. While specific data on ATR expression in M059J cells after α radiation is not provided, studies on other cell types show that ATR is activated in response to DNA damage from ionizing radiation, including α particles^43^. The deficiency in DNA-PKcs in this cell line results in less efficient DSB repair. *XRCC6*, encoding Ku70, is a critical component of the NHEJ pathway, which is essential for repairing DSBs induced by ionizing radiation. Studies have shown that the expression of *XRCC6* can be upregulated in response to ionizing radiation^44^. For instance, in rat hippocampus tissue, *XRCC6* expression was significantly increased after exposure to different doses of radiation, reaching a peak at 6 h post-exposure. Studies on glioblastoma cell lines, including M059J, have shown that γ-H2AX levels increase following exposure to ionizing radiation, indicating DNA damage, which is consistent with strong expression levels of the gene *H2AFX* encoding H2AX^45^. The intensity and duration of γ-H2AX expression can vary depending on the radiation dose and the cell line’s inherent radiosensitivity. It was demonstrated, when M059J cells were exposed to alpha particles, they showed increased recruitment of RAD51, a protein closely related to RAD54L in the homologous recombination repair pathway^35^. The accumulation of RAD51 in M059J cells post-irradiation suggests a heightened activity of homologous recombination due to the deficiency in NHEJ, implying that RAD54L might also be upregulated or more active in these conditions to compensate for the repair deficit. This interpretation is further supported by our γH2AX analysis, which revealed a dose-dependent induction of foci in both cell lines, with M059K accumulating higher numbers of foci, whereas M059J exhibited larger and more clustered radiation-induced foci, consistent with impaired DSB repair kinetics due to DNA-PKcs deficiency. Consistent with their DNA-PKcs status, M059K cells showed a stronger initial γH2AX response and a faster decline in foci between 15 min and 6 h post-irradiation, indicating more efficient DSB repair compared with M059J, whereas the higher γH2AX levels observed in M059K at 24 h may reflect differences in checkpoint recovery and cell-cycle dynamics rather than impaired repair.

To our knowledge, this is the first report integrating direct irradiation of glioblastoma cells with this source and subsequent pathway-focused gene expression analysis. This dual approach not only confirms the technical feasibility and reproducibility of the irradiation system but also provides a broad overview of the DNA damage response in DNA-PKcs–deficient versus proficient glioblastoma cells under high-LET conditions. Beyond its descriptive value, our dataset has translational implications. The strong induction of *ATR*, *H2AFX*, and *RAD54L* in DNA-PKcs–deficient M059J cells indicates compensatory reliance on homologous recombination and checkpoint pathways, which may render such tumors particularly sensitive to ATR inhibitors, PARP inhibitors, or checkpoint blockade in combination with radiotherapy. In contrast, the restricted transcriptional response observed in DNA-PKcs–proficient M059K cells underscores their constitutive NHEJ capacity and suggests that pharmacological DNA-PKcs inhibition could be exploited to radiosensitize such tumors. Together, these findings outline potential therapeutic vulnerabilities and justify further mechanistic validation across a broader panel of glioblastoma models.

M059K cells exhibited a much narrower transcriptional response to irradiation, characterized by the prominent activation of *MSH2* and moderate increases in *XRCC6* and *ATR*. This suggests that mismatch repair contributes to the response to complex DNA lesions beyond its classical role in correcting base mismatches. The strong induction of *MSH2* may reflect the activation of additional DNA damage recognition and signaling mechanisms that cooperate with NHEJ to maintain genome stability under conditions of clustered DNA damage^46^. MSH2 is a crucial protein involved in DNA mismatch repair and has been implicated in the repair of DSBs and base lesions induced by ionizing radiation^47^. Studies have shown that cells deficient in MSH2 exhibit increased sensitivity to ionizing radiation, with impaired DNA repair mechanisms and altered cell cycle checkpoint responses. It was demonstrated that minor changes in *MSH2* expression have a profound impact on the treatment and progression of glioblastoma^46^. *MSH2* serves as a critical mediator of chemotherapeutic agent temozolomide (TMZ) sensitivity, and its expression levels can predict therapeutic outcomes. Understanding the regulation of MSH2 and its role in GBM can help develop better therapeutic strategies and improve patient prognosis. This suggests that DNA-PKcs-proficient cells maintain sufficient basal repair capacity and rely less on transcriptional reprogramming after DNA damage, in line with the role of DNA-PKcs in rapidly resolving double-strand breaks through constitutive NHEJ activity.

When comparing irradiated M059J and M059K cells, only *PRKDC*, *MSH3*, and *ERCC1* remained more highly expressed in M059K, reflecting the underlying DNA-PKcs proficiency and constitutive activation of mismatch repair and nucleotide excision repair pathways. The mismatch repair system, which includes genes like *MLH1* and *MSH2*, plays a critical role in recognizing and repairing DNA damage caused by ionizing radiation^48^. Although *MSH3* is part of the mismatch repair system, the specific studies provided do not mention its expression levels post-radiation. ERCC1 is a DNA repair protein involved in the nucleotide excision repair pathway, which is crucial for repairing radiation-induced DNA damage. Ionizing radiation has been shown to activate *ERCC1* expression in different cell lines, such as prostate carcinoma cells and glioma cell lines, however was not tested in M059 cell lines^49,50^. The overall transcriptional profiles suggest that, in the absence of DNA-PKcs, glioblastoma cells compensate by activating multiple repair mechanisms, while proficient cells utilize pre-existing DNA repair pathways with minimal transcriptional changes.

These findings have potential translational relevance in glioblastoma therapy. DNA-PKcs-deficient tumors, such as those resembling the M059J profile, may be particularly reliant on compensatory pathways like homologous recombination and ATR/CHK checkpoint signaling, making them potentially sensitive to PARP inhibitors, ATR inhibitors, or combined radiotherapy and checkpoint inhibition. In contrast, tumors with intact DNA-PKcs may exhibit primary resistance to DNA-damaging therapies due to constitutive NHEJ activity, suggesting that DNA-PK inhibitors could be used to sensitize such tumors. Overall, profiling DNA repair gene expression and DNA-PKcs status could help stratify glioblastoma patients for personalized radiotherapy and DNA repair-targeting therapies.

## Supplementary Information

Below is the link to the electronic supplementary material.

Supplementary Material 1

## Data Availability

The datasets supporting the conclusions of this article are included within the article.
