# Supplementary material for: Expression of DNA-damage response genes after exposure to high LET particles used in BNCT in glioblastoma cells with altered radiosensitivity
Source: Sci Rep. 2025 Dec 17;16:2822. doi: 10.1038/s41598-025-32635-1 (PMC12824391; doi:10.1038/s41598-025-32635-1)
Supplement: Supplementary file 1 — Supplementary Material 1 [file 41598_2025_32635_MOESM1_ESM.pdf]

Figure S1

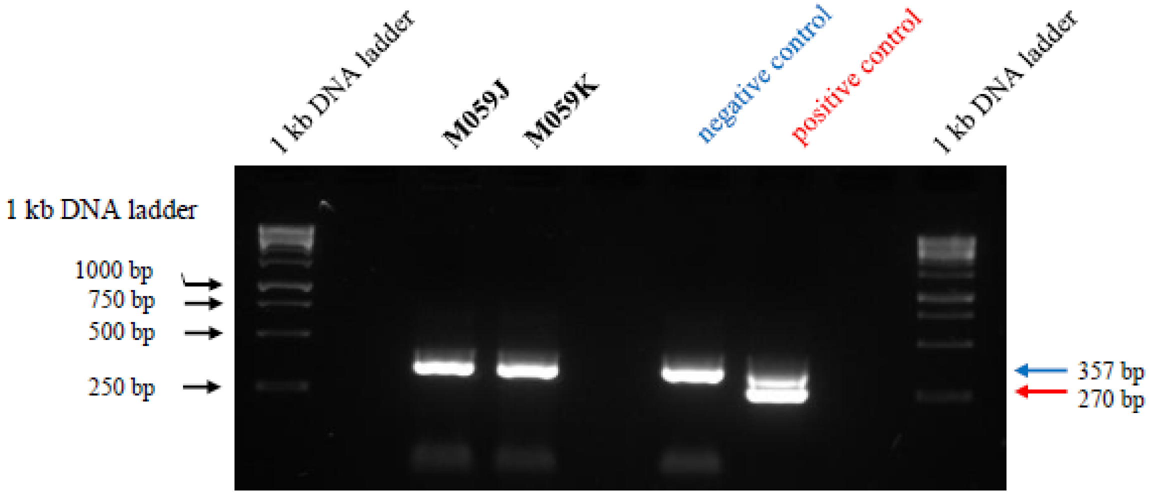

Figure S1. *Mycoplasma* test based on PCR using EZ-PCR Mycoplasma Detection Kit (Cat. No.: 20-700-20). Conditions: 10 µl PCR/lane, 2% agarose, 0.5 x TBE, 100V, 40 min. *Mycoplasma* positive control shows a 270 bp band as well as 357 bp band. *Mycoplasma* negative samples (from M059J and M059K) and negative control shows a 357bp band only. Primer self-annealing yield bands of <100bp in size. Ladder: 1 kb GeneRuler ready-to-use-DNA ladder (Thermo Scientific, SM0313).

Figure S2

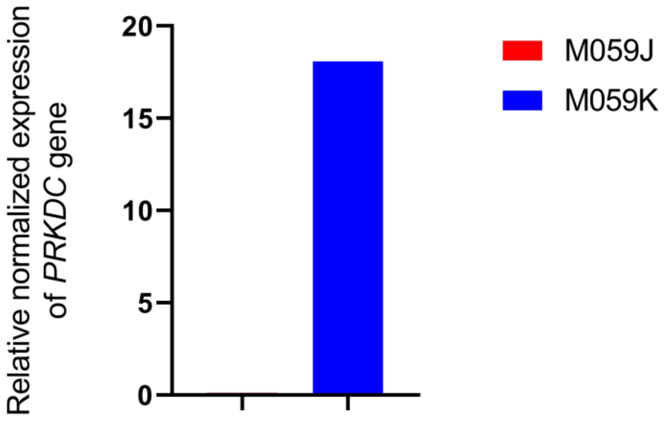

Figure S2. Relative normalized expression of the *PRKDC* gene in glioblastoma cell lines M059J and M059K. Relative mRNA expression of *PRKDC* gene was quantified by qPCR under non-irradiated conditions (0 Gy). Expression levels were normalized to *ACTB* (β-actin) as a reference gene and are presented relative to M059J. M059K cells exhibited *PRKDC* expression compared to M059J cells, consistent with the DNA-PKcs–proficient status of M059K and the known frameshift mutation resulting in DNA-PKcs deficiency in M059J. Bar plot of the relative expression of *PRKDC* gene was calculated *via* the ΔΔCt method. The bars correspond to the mean ± SEM (n = 3).
